# The expanded clinical profile and the efficacy of colchicine therapy in Egyptian children suffering from familial mediterranean fever: a descriptive study

**DOI:** 10.1186/1824-7288-38-66

**Published:** 2012-12-04

**Authors:** Hala Salah El-Din Talaat, Mohamed Farouk Mohamed, Nihal Mohamed El Rifai, Mohamed Ali Gomaa

**Affiliations:** 1 Department of Pediatrics, Faculty of Medicine, Cairo University, Cairo, Egypt; 2 Al Mounira Hospital, Cairo, Egypt; 3 New University Children’s Hospital (Abu El Reish), 4 – Gamal Salem St. Doki, Cairo, Egypt

**Keywords:** Familial Mediterranean fever, Clinical presentations, Efficacy, Colchicine

## Abstract

**Background:**

Familial Mediterranean fever (FMF) is an autosomal recessive disease characterized by self-limiting recurrent attacks of fever and serosal inflammation, leading to abdominal, thoracic or articular pain.

**Objective:**

To detect variable clinical presentations and genotypic distribution of different groups of FMF patients and the efficacy of colchicine therapy in treatment of these groups of FMF after one year.

**Methods:**

A cross-sectional study was conducted on 70 patients already diagnosed with FMF and following-up at the Rheumatology Clinic, Children's Hospital - Cairo University. Diagnosis of FMF was determined according to Tel Hashomer criteria for FMF. All patients were subjected to a questionnaire including detailed history with emphasis on clinical manifestations and colchicine dose to control attacks. Mutational analysis was performed for all study subjects covering 12 mutations in the MEFV gene: E148Q, P369S, F479L, M680I (G/C), M680I (G/A), I692del, M694V, M694I, K695R, V726A, A744S and R761H. Response to colchicine treatment was evaluated as complete, incomplete and unresponsive.

**Results:**

Out of the 70 patients- 40 males and 30 females- fever was the most common presenting feature, followed by abdominal pain, and arthritis; documented in 95.7%, 94.3%, and 77.1% of cases respectively. Mutational analysis detected gene mutation on both alleles in 20 patients (homozygotes), on only 1 allele in 40 patients (heterozygotes), and on none of the alleles (uncharacterized cases). Mild to moderate disease severity score (according to Tel Hashomer key to severity score) was detected in a significant proportion of heterozygotes and the uncharacterized group than the homozygotes. All patients received colchicine therapy; 22.9% of them showed complete response, 74.3% showed incomplete response and 2.9% showed no response to therapy. The colchicine dose needed to control attacks was significantly lower in heterozygotes than the homozygotes(P=0.04). Also patients’ response to colchicine therapy was significantly better in the heterozygous group(P=0.023).

**Conclusion:**

Fever, abdominal pain and arthritis are the most common presenting features for homozygous, Heterozygous and uncharacterized patients. E148Q, V726A, and M680I were the most common mutations detected in the heterozygous group. Homozygosity were found for M680I, M694V, and M694I mutations in 13 patients (65% of homozygotes). Heterozygotes presenting with severe phenotype should be further analyzed for less common second MEFV mutation using gene sequencing. The colchicine dose required to control the attacks was significantly lower and patients’ response to colchicine therapy was significantly better in the heterozygous group than homozygous group.

## Introduction

FMF is an autosomal recessive disease characterized by self-limiting recurrent attacks of fever and serosal inflammation, leading to abdominal, thoracic or articular pain. Erysipelas-like skin lesions and diffuse myalgia are less frequent manifestations [1]. The disease course can be complicated by development of amyloid deposition and organ failure which can be fatal [2].

The frequency of FMF in any location depends on the ethnic background of the population. Specific ethnic groups have an increased prevalence [3]. The disease is most prevalent among non-Ashkenazi Jews, Arabs, Turks and Armenians with carrier frequencies of 1:5 to 1:16, 1:5, 1:5, and 1:7, respectively [4].

The diagnosis of FMF has been based before on the clinical features and exclusion of other causes of periodic fever, thus making it difficult to establish a correct diagnosis in patients with milder or atypical manifestations of the disease [5].

Evidences accumulating suggest that the clinical spectrum of FMF associated with MEFV mutations extends from the typical manifestation of the full blown disease to the asymptomatic state, this make the molecular analysis of MEFV a useful tool in the clinical practice [6]. In the clinical context of FMF, the presence of two mutations on different alleles (homozygousity or compound heterozygousity) makes it possible to confirm the diagnosis of FMF but when only one mutation is present, the diagnosis is not ascertained, but neither can it be excluded [7].

The goals of therapy are to reduce morbidity and to prevent complications of the disease. Treatment of FMF at this point consists of taking colchicine, a neutrophil-suppressive agent. Colchicine is so effective in preventing attacks of FMF and the development of amyloidosis that the most important aspect of medical care is to make the correct diagnosis and to institute therapy [8].

This study aimed to detect variable clinical presentations and genotypic distribution of FMF patients and the efficacy of colchicine therapy in treatment of these groups of FMF after one year.

## Methods

A cross-sectional study was conducted on 70 patients with FMF during the period from January, 2011 to July, 2011. The diagnosis of FMF was determined according to Tel Hashomer criteria for FMF shown in Table 1.

**Table 1:** **Tel Hashomer criteria for familial Mediterranean fever**[1]

**Major criteria**	**Minor criteria**
· Recurrent febrile episodes accompanied by serositis	· Recurrent febrile episodes
· Amyloidosis of AA-type without a predisposing disease	· Erysipelas-like erythema
· Favorable response to continuous colchicine treatment	· Familial Mediterranean fever in a first-degree relative

*If 2 major Tel Hashomer criteria or one major criterion and two minor ones are satisfied the diagnosis of FMF can be confirmed; if only one major and one minor criteria are satisfied the diagnosis is only probable.

The Scientific Research Committee of Pediatrics Department, Faculty of Medicine-Cairo University, approved the study design. All data were confidential for the research use only.

Patients fulfilled the criteria of being diagnosed with FMF for more than one year and were followed up and adherent to treatment in the Rheumatology clinic, Children's Hospital, Cairo University for more than one year and those diagnosed for less than one year and non-compliant patients either to therapy or to follow up visits were excluded.

All patients were subjected to a questionnaire containing detailed history, demographic status (gender, age of onset, age of diagnosis, time interval between disease onset and diagnosis, duration of follow-up, number of attacks per year before and after colchicine treatment, consanguinity, family history of FMF, diseases that may be associated with FMF, colchicine dosage to control attacks, clinical manifestations (fever, abdominal pain, arthritis, chest pain, and erysipelas-like erythema), history of other diseases and development of amyloidosis, presence of a family history of FMF, amyloidosis and a treatment regimen was developed. Then, Response to colchicine treatment was evaluated as complete (attack free), incomplete (decline >50% in the frequency of attacks) and unresponsive.

According to clinical condition, the severity score of the disease was calculated based on the Tel Hashomer Severity Score shown in Table 2.

**Table 2:** **Tel Hashomer key to severity score**[9]

	***Points***
***Age of onset***	
< 5 years	3
5–10 years	2
10–20 years	1
> 20 years	0
***Frequency of attacks***	
> 2 per month	3
1–2 per month	2
< 1 per month	1
***Colchicine dosage to control attacks***	
Nonresponders	4
2 mg/day	3
1.5 mg/day	2
1 mg/day	1
***Arthritis***	
Protracted arthritis	3
Presence of acute joints	2
Erysipelas-like erythema	2
Amyloidosis	3
Phenotype II	4

Total of points indicates the following: mild disease, 2–5 points; moderate disease, 6–10 points; severe disease, >10 points.

### Mutation analysis

The FMF gene mutations were tested using the FMF Strip AssayTM, ViennaLab Labordiagnostika GmbH, Vienna, Austria. The assay is based on polymerase chain reaction (PCR) and reverse hybridization and includes three successive steps: (1) DNA isolation from anticoagulated blood, (2) PCR ampliWcation using biotinylated primers, (3) hybridization of amplifcation products to a test strip containing both wild and mutant allele-specific oligonucleotide probes immobilized as an array of parallel lines. Bound biotinylated sequences were detected using streptavidin–alkaline phosphatase and color substrates. The assay covers 12 mutations in the MEFV gene: E148Q, P369S, F479L, M680I (G/C), M680I (G/A), I692del, M694V, M694I, K695R, V726A, A744S and R761H [10].

For each polymorphic position, one of three possible staining patterns were obtained either wild type probe only (normal genotype), wild type and mutant probe (heterozygous genotype or carrier) or mutant probe only(homozygous mutant genotype or affected). Records of patients were classified into three groups according to the presence of gene mutation on both of the alleles (homozygotes), on only 1 allele (heterozygotes), and on none of the alleles (uncharacterized group).

Urine analysis were also collected to detect the presence of any abnormalities especially proteinuria or hematuria.

### Ethical considerations

The aim and nature of the study was explained for each candidate and/or parent before inclusion. An informed written consent was obtained from parents/surrogates before enrollment. Children old enough were asked for consent.

### Statistical analysis

Data were statistically described in terms of range, mean ± standard deviation (± SD), median, frequencies (number of cases) and percentages when appropriate. Comparison of numerical variables between the study groups was done using Kruskal Wallis test with Mann Whitney *U* test for independent samples as posthoc multiple 2-group comparisons. For comparing categorical data, Chi square (χ^2^) test was performed. Exact test was used instead when the expected frequency is less than 5. *p* values less than 0.05 was considered statistically significant. All statistical calculations were done using computer programs Microsoft Excel 2007 (Microsoft Corporation, NY, USA) and SPSS (Statistical Package for the Social Science; SPSS Inc., Chicago, IL, USA) version 15 for Microsoft Windows.

## Results

Patients were classified according to mutations and allele status into 3 groups: group 1 (homozygous group) included 20 patients (28.6%) of whom 12 were males and 8 were females (M:F = 1.5:1), group 2 (heterozygous group) included 40 patients (57.1%) of whom 21 were males and 19 were females (M:F = 1.1:1) and group 3 (uncharacterized patients) included 10 patients (14.3%) of whom 7 were males and 3 were females (M:F = 2.3:1).

The mean age of onset and mean age at diagnosis of all study population, were 4.86 ± 2.56, and 7.14 ± 2.89 years respectively. The mean time interval between disease onset and diagnosis was 2.31 ± 1.57 years ranging from 0.6 – 7 years. The duration of follow-up period was ranging from 1–11 years with a mean of 2.75 ± 2.35 years.

Comparing patients’ characteristics, the mean age at diagnosis was significantly higher in heterozygous than homozygous group (*P=0.02)*. Also, the mean duration of follow-up was significantly lower in the heterozygous patients *(P=0.03)*. However, no statistically significant differences were detected between heterozygous and the uncharacterized group regarding the above mentioned parameters. Moreover, none of the above mentioned parameters were statistically significant when comparing patients’ characteristics, consanguinity and family history rates of all study groups (Table 3).

**Table 3:** Comparison between patients’ characteristics, consanguinity and family history rate of the three groups

**Item**	**Heterozygous (n=40)**	**Homozygous (n=20)**	**Gene-ve (n=10)**	**P-value**
Age of onset (yeas), mean ± SD	5.20 ± 2.51	4.32 ± 2.58	4.63 ± 2.76	0.35
Age at diagnosis (years), mean ± SD	7.81 ± 2.79	6.01 ± 2.75	6.76 ± 3.08	0.07
Time interval between disease onset and diagnosis (years)				
Mean ± SD	2.63 ± 1.63	1.75 ± 1.03	2.15 ± 1.94	0.13
Median (range)	2 (1–6)	1.8 (1–4)	1.3 (1–7)	
Duration of follow-up (years), mean ± SD	2.18 ± 1.41	3.80 ± 3.24	2.95 ± 2.77	0.11
Median (range)	1.75 (1–6)	2 (1–11)	1.95 (1 – 9)	
Male/Female	21/19	12/8	7/3	0.58
Consanguinity (n%)	14 (35%)	11 (55%)	1 (10%)	0.05
Family history of FMF (n%)	7 (17.5%)	7 (35%)	0 (0%)	0.06
Family history of amyloidosis (n%)	4 (10%)	0 (0%)	0 (0%)	0.20

FMF*=* familial Mediterranean fever.

*P-value less than 0.05 is considered statistically significant.

**Figure 1:**
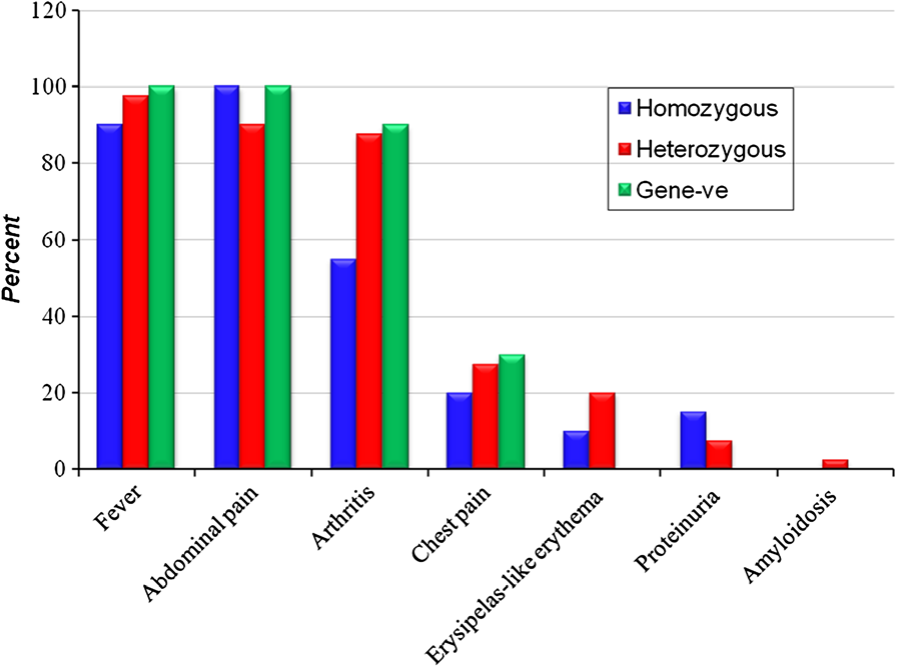
Clinical profile of the different study groups.

As regards the clinical features of all patients; fever was the most common presenting feature in 95.7% of cases (67 out of 70 patients), abdominal pain was a constant feature in both homozygotes and uncharacterized groups and was documented in all patients of the two groups. Arthritis was a common feature in both heterozygotes and the uncharacterized group and was documented in 87.5% (35/40), and 90% (9/10) of patients respectively. Proteinuria was detected in 6 patients (8.6%) in whom renal biopsy was indicated. Amyloidosis was detected in only one heterozygous case; diagnosis was based on the result of renal biopsy (Figure 1).

Regarding response to colchicine therapy, it was complete in 16/70 (22.9%), incomplete in 52/70 (74.3%)and no response in (2.9%). Although incomplete response was documented in a significant proportion of patients, the mean number of attacks per year was significantly lower after treatment (4.51 ± 4.82, range=0 - 24) than before initiation of therapy *(*24.09 ± 10.02, range=10-72); *P=0.00*.

Table 4 shows that the disease severity score was moderate in all of the uncharacterized patients (100%). The mild to moderate disease severity score was noted in a significant proportion of heterozygous and gene-negative groups than homozygous group *(P=0.01)*. Also patients’ response to colchicine therapy was significantly better in the heterozygous group (*P=0.023*). Moreover, the mean colchicine dose that was required to control the attacks was lower in the heterozygous group than homozygous and gene –ve groups and that difference was statistically significant *(P=0.04)*.

**Table 4:** Comparison between disease severity score, response to colchicine therapy, and number of attacks per year in the three study groups

**Item**		**Hetero-zygous (n=40)**	**Homo-zygous (n=20)**	**Gene-ve (n=10)**	**P-value**
Disease severity score, n (%)	Mild	9 (22.5)	0 (0.0)	0 (0.0)	0.01*
	Moderate	26 (65.5)	14 (70)	10 (100)	
	Severe	5 (12.5)	6 (30)	0 (0.0)	
Response to colchicine, n (%)	Complete	13 (32.5)	1 (5.0)	2 (20)	0.09
	Incomplete	25 (62.5)	19 (95.0)	8 (80)	
	No response	2 (5)	0 (0.0)	0 (0.0)	
Colchicine dose to control attacks, mean ± SD		1.26 ± 0.36	1.47 ± 0.34	1.40 ± 0.21	0.04*
Number of attacks per year, mean ± SD	Before treatment (range)	23.70 ± 11.47 (10–72)	24.40 ± 6.95 (12–36)	25 ± 9.76 (16–48)	0.6
	After treatment (range)	4.80 ± 5.81 (0–24)	3.95 ± 2.50 (0–10)	4.5 ± 4.22 (0–12)	0.9

*P-value less than 0.05 is considered statistically significant.

**Figure 2:**
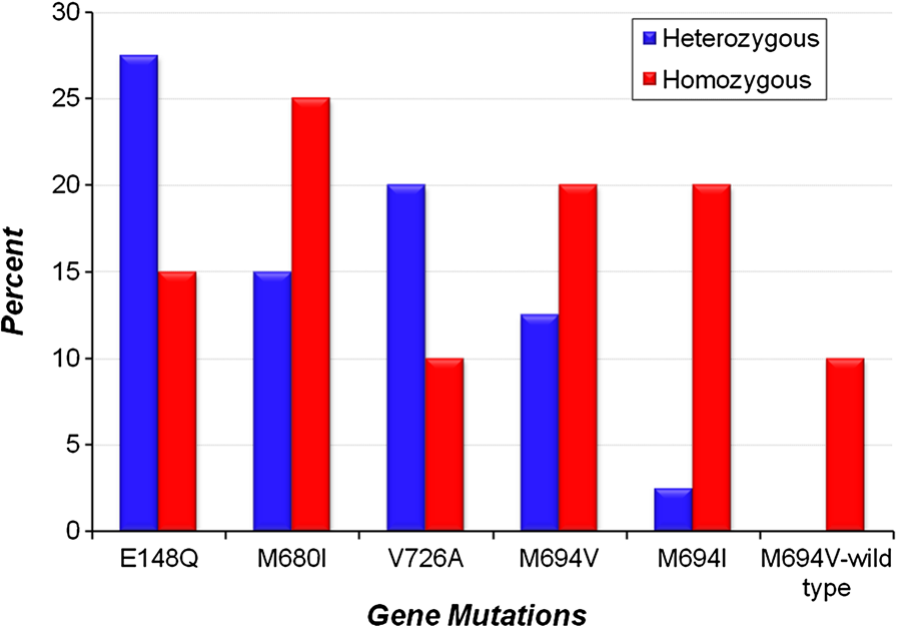
Major genotypes in heterozygous and homozygous groups.

Figure 2 demonstrates the major genotypes in both heterozygous and homozygous groups. As shown, E148Q, V726A, and M680I mutations were the most common mutations seen in the heterozygous group and were found in 11/40 patients (27.5%), 8/40 patients (20%), and 6/40 patients (15%) respectively. Homozygosity for M680I, M694V, and M694I were found in 5/20 patients (25%), 4/20 patients (20%), and 4/20 patients (20%) respectively.

In the homozygous group, out of 20 patients, diseases associated with FMF were documented in 4 patients (20%); Henoch-schonlein purpura (HSP) in one patient (5%) with M694V-wild type mutation, inflammatory bowel disease (IBD) in two patients (10%) with M694V and M680I mutations, SLE (systemic lupus erythematosus) in one patient (5%) with M680I mutation. Regarding the heterozygous group, out of 40 patients, diseases associated with FMF were documented in 9 patients (22.5%); HSP in two patients (5%) with E148Q and M680I mutation, IBD in one patients (2.5%) with V726A mutation, JRA (juvenile rheumatoid arthritis) in three patients (7.5%) with E148Q, M680I and M694V-V726A mutations, SLE in two patients (5.0%) with V726A and E148Q mutations, Rheumatic fever in one patient (2.5%) with M694V mutation. Regarding mortality rate, none of our patients had died during the period of the study.

## Discussion

FMF is an autoinflammatory disease common in eastern Mediterranean populations. It is characterized by febrile episodes of serosal and synovial inflammation causing marked increase in the acute phase response [11]. FMF is more prevalent in males, with a male to female ratio of 1.5-2:1 [12].

In the current study, a male preponderance was noted in all the study population, with an overall M:F ratio of 1.3:1. Male to female ratios of 1.5:1, 1.1:1, and 2.3:1 were reported among homozygotes, heterozygotes, and the uncharacterized group respectively. A male preponderance was noted in different other studies. El-Garf et al. [13], who studied 136 Egyptian patients in the period between January, 2005 and July, 2008 recruited from the rheumatology clinic, Pediatric Hospital, Cairo University, as well as three referral centers (a general rheumatology, pediatric rheumatology and general pediatric clinics), also reported a M:F ratio of 1.9:1. Similarly, Settin et al. [14], who studied 66 Egyptian patients who were referred from various hospitals to Genetics Department, Mansoura University, Children’s Hospital for confirmation of diagnosis through molecular analysis also reported a M:F ratio of 1.3:1. Another retrospective study done by Booty et al. [15] reported that out of 28 FMF patients with only one identified MEFV mutation, and who were seen at the National Institutes of Health, Bethesda MD - USA, 15 were males and 13 were females with M:F ratio of 1.15:1.

However, Duşunsel et al. [16] who reviewed the medical records of 102 Turkish patients, documented a M:F ratio of 1:1.3 (a slight female preponderance), but these results are not statistically significant and do not support the suggestion that FMF may have incomplete penetrance in female subjects [17]. Although a male predominance among FMF patients has been documented in several ethnic groups, most studies have reported that FMF affects both genders in a similar ratio [18,19].

The onset of clinical manifestations in FMF occurs before 5 years of age in 63–68% of cases and before 20 years of age in 90% of cases. The onset may be as early as 6 months of age [20]. In our study, the mean age of onset, mean age at diagnosis, and mean time interval between disease onset and diagnosis of all study population, were 4.86 ± 2.56, 7.14 ± 2.89, and 2.31 ± 1.57 years respectively. The mean age at diagnosis was significantly higher in heterozygous group than homozygous group and the mean duration of follow-up was significantly lower in the heterozygous patients. Our study populations had age of onset much earlier than reported. In a retrospective study done by Ebru [21] that was carried out on 415 clinically diagnosed FMF Turkish patients; the mean age of onset was 13.9 ± 9.8 years. Duşunsel et al. [16] also reported a mean age of onset, a mean age at diagnosis, and a median time interval between disease onset and diagnosis of 6.8 ± 3.7, 9.7 ± 3.7 and 2 (0.5–11) years respectively. This earlier age of onset that was observed in our study may be explained by the early detection of FMF patients in our rheumatology pediatric unit by clinical suspicion before confirmation by genetic analysis.

FMF is considered as an autosomal recessive hereditary disease, associated with a single gene named MEFV [22]. However, about one-third of FMF patients bear a single mutation on one allele, suggesting that the disease might be transferred as an autosomal dominant trait with partial penetration. Alternatively, an additional gene—yet to be identified—might be responsible for the disease in these cases with single allele mutation [23].

The inherited pattern of FMF which is mostly recessive was supported on the basis of consanguinity and positive family history. Regarding consanguinity of the study population; 37.14% of patients had consanguineous parents, all of them were homozygotes and heterozygotes except one patient who was gene-negative. A positive family history of FMF was recorded in 14 patients (20%), of whom 7 patients were homozygotes and 7 patients were heterozygotes. Family history of amyloidosis was noted in 4 patients (5.7%) all of them were heterozygous. Consanguinity in our study is nearly similar to Duşunsel et al. [16] who reported that 30.4% of their patients had consanguineous parents, a positive family history of FMF was recorded in 26.5% of patients and a family history of amyloidosis was noted in 5.9% of patients. El-Garf et al. [13] reported that, out of their 136 patients, a positive consanguinity was present in 32 (23.5%) patients and a positive family history in 45 (33.1%) patients. Settin et al. [14] showed that 21.2% of their patients had a positive family history for FMF; however parental consanguinity was positive in 63.3% of these cases which was more than that in our results. These differences may due to familial predisposition of FMF in certain populations and the impact of cultural traditions.

Regarding clinical presentations among our study groups; fever was a prevalent feature in all groups, and was documented in 90%, 97.5%, and 100% of homozygtes, heterozygotes, and the uncharacterized group respectively. Abdominal pain was a constant feature in both homozygotes and the uncharacterized group and was documented in all patients. Arthritis was a common feature in both heterozygotes and the uncharacterized group and was documented in 87.5%, and 90% of patients respectively. Variation in clinical picture of FMF patients with positive gene mutation was in agreement with many reports but differ from each other in the frequency of each clinical picture; this difference may be due to the difference in racial group and geographic region, and may be due to the difference in predominant gene mutation in different populations (phenotype genotype correlation) [24,25].

Based on the Tel Hashomer Severity Score [10], severity score of the disease was calculated. The mean severity score was 8.27 ± 2.03. Mild to moderate disease severity scores were detected in a significantly higher proportion of heterozygotes and the uncharacterized group than homozygotes.

Attacks of FMF can be prevented by prophylactic colchicine (0.02-0.03 mg/kg/day; maximum: 2 mg/day) in 1 to 2 divided doses. Colchicine therapy reduces the frequency of acute attacks, but also greatly decreases the probability of development of amyloidosis; it may produce partial regression of existing amyloidosis [20]. In our study all patients received colchicine therapy (dose 0.5 - 2 mg/day); only 22.9% of them showed complete response, 74.3% showed incomplete response and 2.9% showed no response. Although incomplete response to colchicine was documented in a significant proportion of patients, the mean number of attacks per year was significantly lower after treatment than before initiation of therapy. Colchicine dose needed to control attacks was significantly lower in heterozygotes than homozygotes. Also response to colchicine therapy was significantly better in the heterozygous group. Contrary to our results, Al-Wahdneh and Dahabreh [26] reported that prescribing colchicine to all of their patients, in doses ranged between 0.5 mg and 2 mg daily according to age and response, resulted in disappearance of the attacks completely in 68% of cases, a significant decrease in the number and severity of attacks in 29% and no response to treatment in 3% of patients. Similarly, Duşunsel et al. [16] reported that 77.5% of their patients had complete response, 13.7% had some attacks despite colchicine, and 2% were unresponsive. Booty et al. [15] also reported that among their 28 heterozygous patients, information on colchicine responsiveness was unavailable for 3 patients, colchicine response was either complete or partial in 84% of patients (21/25), and 4 patients did not respond at all. Fourteen patients (56%) had a complete response, while 7 patients (28%) had periodic attacks of inflammation although less frequently while on treatment. The reasons for lacking response to colchicine could be explained by knowing the fact that colchicine has to go through several stages on its way to controlling inflammation, therefore its efficacy may be affected at various points. Theoretically, problems with its absorption in the intestine can change the therapeutic plasma levels. Problems with the functioning of the human multidrug resistance (MDR1 gene) (P-glycoprotein pump) in white blood cells or serous membrane cells can also affect colchicine function [27,28]. Modulation of colchicine metabolism by different factors (erythromycin, clarithromycyn, lovastatin, simvastatin, cyclosporin, grapefruit juice, etc.) at the level of cytochrome 3A4 can also influence the effect of colchicine [29].

In our study, out of 70 patients, 5 patients (7.1%) underwent surgery; appendectomy was performed in 4 patients and herniotomy was done in only one patient. Our results are nearly similar to Al-Wahadneh and Dahabreh [26] who reported that only 2 out of 56 patients (3.7%) underwent appendectomy. Duşunsel et al. [16] also reported that 4 out of 102 patients (3.4%) underwent surgery, and all of them had appendectomy. Settin et al., [14] reported a higher percent of patients who underwent surgery; 15 out of 66 patients (22.7%) underwent laparotomy during severe abdominal pain either for exploration or for appendectomy. This high percent could be a result of the severe abdominal pain that forced the patients to be bed ridden and that was documented in 66.7% of their patients.

In our study, the most frequent mutations in all patients were E148Q, M680I, and V726A and were detected in 20%, 15.7%, and 14.3% of patients respectively. In an Egyptian study, among 66 patients, M694V was the most common allelic mutation found followed by V726A then M680I (18.8%, 17.42% 12.1% respectively) [14]. In another Egyptian study, among 136 patients, it was found that the most frequent gene mutations were V726A, M694V, M680I, E148Q and M694I in 41.2, 32.4, 29.4, 25 and 20.6% of patients respectively [13]. This differences in the allelic mutations found among patients could indicate the mutational heterogeneity of FMF in the Egyptian population. This mutational heterogeneity appears to be less obvious among other ethnic populations. M694V mutation was found in 97% of the North African Jews in Israel [30], while M694I mutation was present in 80% of Algerian Arabs [31]. In the studies by Touitou et al. [6], and by the Turkish FMF study group [11], the most common MEFV mutation in Turkey is M694V (57.0 and 51.4%, respectively), followed by M680I (16.5 and 14.4%, respectively), and V726A (13.9 and 8.6%, respectively). Possible explanation is that having only one MEFV mutation may give rise to a FMF phenotype in the presence of one or more modifying alleles in other related genes, or other environmental factors like a stress. Asymptomatic carriers for one FMF mutation have biochemical evidence for subclinical inflammation [7,32] and a more recent study found a higher frequency of carriers for highly penetrant FMF mutations among patients with systemic inflammatory response syndrome (SIRS) and sepsis [33]. Therefore, modifying alleles could contribute to an inflammation dosage threshold, which is necessary to develop systemic inflammation and symptomatic FMF.

HSP and polyarteritis nodosa (PAN) are more common in FMF patients than in the general population [19]. In our study, HSP was determined in 4.3% of patients and this in agreement with a high prevalence of vasculitis found in FMF patients but PAN was not found. Duşunsel et al. [16] also reported HSP in 6.8% of their patients.

## Conclusion and recommendations

We confirm that in the current study of 70 patients with FMF; fever, abdominal pain and arthritis were the most common presenting features for homozygous, heterozygous and uncharacterized patients. E148Q, V726A, and M680I were the most common mutations detected in the heterozygous group. Homozygosity were found for M680I, M694V, and M694I mutations in 13 patients (65% of homozygotes). The mean colchicine dose required to control the attacks was significantly lower and patients’ response to colchicine therapy was significantly better in the heterozygous group than homozygous group.

Wide indications for genotyping based on expanded clinical profile led to more frequent diagnosis of FMF. Concerning heterozygotes presenting with severe phenotype, they should be further analyzed for less common second MEFV mutation using gene sequencing. Even the 10 uncharacterized patients with positive FMF criteria -according to Tel Hashomer criteria for diagnosis of FMF- who had no identified mutations, the following explanations could account for this observation; we screened only for 12 set of genes despite of presence of 160 gene mutation update. Secondly, unknown mutation may exist. Thirdly another as-yet-unidentified FMF locus may exist. So, despite the current knowledge regarding FMF, prospective clinical studies with large numbers of patients and different ethnic groups will help to better clarify this considerable disease.

## Abbreviations

FMF: Familial mediteranian fever; HSP: Henoch-schonlein purpura; IBD: Inflammatory bowel disease; SLE: Systemic lupus erythematosus; JRA: Juvenile rheumatoid arthritis.

## Competing interests

The authors declare that they have no competing interests.

## Authors’ contributions

MFM and MAG- Designed, conducted and analyzed the study, HST and NMR- Analyzed the data and drafted the manuscript. All authors have revised and approved this manuscript.

## Funding

The study was self-funded by authors.
